# Phylogenomic Reappraisal of Fatty Acid Biosynthesis, Mycolic Acid Biosynthesis and Clinical Relevance Among Members of the Genus *Corynebacterium*

**DOI:** 10.3389/fmicb.2021.802532

**Published:** 2021-12-23

**Authors:** Lynn G. Dover, Amy R. Thompson, Iain C. Sutcliffe, Vartul Sangal

**Affiliations:** Faculty of Health and Life Sciences, Northumbria University, Newcastle upon Tyne, United Kingdom

**Keywords:** *Corynebacterium*, fatty acid chains, mycolic acid biosynthesis, phylogenomic diversity, virulence

## Abstract

The genus *Corynebacterium* encompasses many species of biotechnological, medical or veterinary significance. An important characteristic of this genus is the presence of mycolic acids in their cell envelopes, which form the basis of a protective outer membrane (mycomembrane). Mycolic acids in the cell envelope of *Mycobacterium tuberculosis* have been associated with virulence. In this study, we have analysed the genomes of 140 corynebacterial strains, including representatives of 126 different species. More than 50% of these strains were isolated from clinical material from humans or animals, highlighting the true scale of pathogenic potential within the genus. Phylogenomically, these species are very diverse and have been organised into 19 groups and 30 singleton strains. We find that a substantial number of corynebacteria lack FAS-I, i.e., have no capability for *de novo* fatty acid biosynthesis and must obtain fatty acids from their habitat; this appears to explain the well-known lipophilic phenotype of some species. In most species, key genes associated with the condensation and maturation of mycolic acids are present, consistent with the reports of mycolic acids in their species descriptions. Conversely, species reported to lack mycolic acids lacked these key genes. Interestingly, *Corynebacterium ciconiae*, which is reported to lack mycolic acids, appears to possess all genes required for mycolic acid biosynthesis. We suggest that although a mycolic acid-based mycomembrane is widely considered to be the target for interventions by the immune system and chemotherapeutics, the structure is not essential in corynebacteria and is not a prerequisite for pathogenicity or colonisation of animal hosts.

## Introduction

*Corynebacterium* is a diverse genus that encompass multiple species of industrial, medical or veterinary importance (Bernard and Funke, 2015; Oliveira et al., 2017; Sangal and Burkovski, 2020). A number of corynebacterial species are commensals (Brugger et al., 2016; Treerat et al., 2020) but some are notable pathogens including the human pathogen *Corynebacterium diphtheriae* and *Corynebacterium pseudotuberculosis*, which is not only primarily pathogenic to sheep but can also infect other animals (Tauch and Burkovski, 2015). *Corynebacterium ulcerans* is a zoonotic pathogen, often acquired by humans from canine pets (Sangal et al., 2014). More recently, several new *Corynebacterium* species that are pathogenic to humans or animals have been identified (Oliveira et al., 2017; Möller et al., 2020; Boxberger et al., 2021; Saunderson et al., 2021). *Corynebacterium glutamicum* is an industrially important member of the genus that is used in large-scale production of several amino acids and aromatic compounds (Ikeda and Takeno, 2013; Kallscheuer and Marienhagen, 2018).

Corynebacteria are Gram-positive bacteria with a complex cell envelope architecture, where corynomycolates (short-chain α-branched, β-hydroxy fatty acids) are esterified to an arabinogalactan polysaccharide that is, in turn, covalently bound to the peptidoglycan cell wall core, forming a mycolyl-arabinogalactan–peptidoglycan complex (Dover et al., 2004; Marrakchi et al., 2014; Burkovski, 2018). These cell-bound mycolic acids form the basis of the inner leaflet of a distinctive outer ‘mycomembrane’, that is completed by intercalation with mycolic acid-containing glycolipids based on trehalose, and other free lipids (Puech et al., 2001; Marchand et al., 2012; Laneelle et al., 2013; Vincent et al., 2018). Thus, the presence of mycolic acids in the cell envelope is associated with stress resistance and pathogenicity both in *Corynebacterium* and *Mycobacterium* strains (Moreira et al., 2008; Vander Beken et al., 2011; Nataraj et al., 2015; Tauch and Burkovski, 2015).

Most knowledge of the roles of mycolic acids in virulence is based on the studies of the human pathogen, *Mycobacterium tuberculosis*. Mycolic acids are involved in the formation of biofilms, affect susceptibility to antibiotics and, in the mycobacteria, play important roles in manipulating the host immune system during the infection (Korf et al., 2005; Dao et al., 2008; Vander Beken et al., 2011; Marrakchi et al., 2014; Nataraj et al., 2015; Batt et al., 2020). T cells specific to mycolic acid offer protection from *M. tuberculosis* infection (Zhao et al., 2015) and an absence of mycolic acid in the cell attenuates the pathogen and modulates cytokine production (Dao et al., 2008; Barkan et al., 2012). However, it should be noted that the size and structural complexity of the mycobacterial mycolic acids is significant here; although the short-chain mycolic acids possessed by corynebacteria can be bound by the CD1 antigen presentation system, they may not activate T-cells (Moody, 2017).

**Figure 1:**
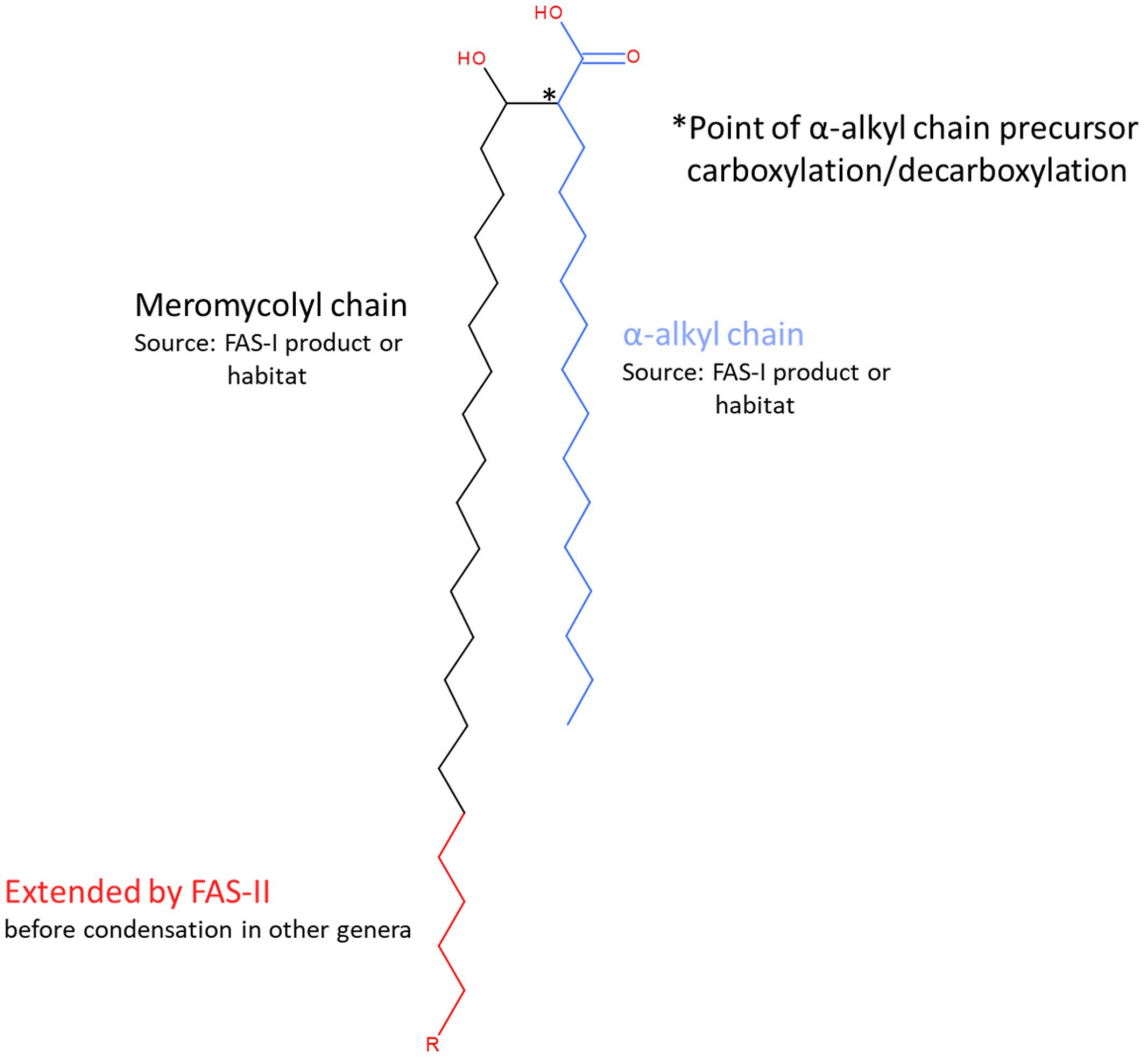
Structural features and sources of mycolic acid components. Mycolic acids are α-alkyl, β-hydroxy branched fatty acids that form the basis of the outer wall permeability barrier or ‘mycomembrane’ that is the defining characteristic feature of the cell wall model originally proposed by Minnikin (1982) for the mycobacteria and other genera that belong to the order *Mycobacteriales*. Briefly, mycolic acids are generated from the condensation of two fatty acyl chains, known as α-and meromycolyl chains. The α-chain (blue) precursor is activated by carboxylation (position indicated by * and see Figure 5). It then participates in a decarboxylative condensation reaction with the carboxylate group of the meromycolyl chain, the residue of which forms a β-keto group that is subsequently reduced to form the characteristic β-hydroxy group of the mature mycolic acid. There are two potential sources of the fatty acid components of mycolic acids. The bacterium may synthesise them *de novo* using fatty acid synthase I or acquire them from its habitat. Corynebacteria produce relatively short corynomycolic acids; fatty acids of approx. 16 C chain lengths are used to form both branches. However, in most other genera in *Mycobacteriales*, the meromycolyl chain is extended by fatty acid synthase II (red chain; the length of R is highly variable but distinctive for each genus and may also contain various structural modifications including double bonds, cyclopropane rings, hydroxy, methoxy, epoxy, wax ester and ketone groups).

Although all mycolic acids are α-alkyl-β-hydroxy fatty acids, those in *Corynebacterium* species (corynomycolates) differ structurally from those of *Mycobacterium* (Dover et al., 2004; Marrakchi et al., 2014). These corynomycolates have 22–36 carbon atoms in total with short α-branch chains (C_8_–C_18_) whereas the total carbon atoms in mycobacteria varies between 60 and 90 with longer chain (C_22_–C_24_) α-alkyl branches and more complex chain modifications (Marrakchi et al., 2014) of the extended meromyoclate chain (Figure 1).

Fatty acids are the direct precursors of mycolic acids and the differences in the length of the meromycolate chains are attributed to the variation in the fatty acid biosynthesis pathways between the two genera. Mycobacterial meromycolate synthesis involves two fatty acid synthases, a eukaryotic-like multifunctional FAS-I that produces a bimodal population of fatty acids of chain lengths C_16_–C_18_ or C_24_–C_26_ (Marrakchi et al., 2014), and a second bacterial-like FAS-II, that elongates these fatty acids to form meromycolate chains (Dover et al., 2004, 2007; Marrakchi et al., 2014). Only FAS-I has been reported in corynebacteria (Dover et al., 2004, 2007; Marrakchi et al., 2014). However, two functional copies of FAS-I, *fasA* and *fasB*, have been reported in *Corynebacterium glutamicum* and *Corynebacterium efficiens* (Stuible et al., 1997; Radmacher et al., 2005).

Given the importance of mycolic acid in the biology of the corynebacteria and the cell envelope in host-bacterium interactions, we have investigated the conservation of genes involved in mycolic acid biosynthesis across the genus *Corynebacterium*. We also attempted to establish an association between the mycolic acid biosynthetic capacities and clinical relevance of the corynebacterial strains.

## Materials and Methods

### *Corynebacterium* Genomes

The genome sequences of 140 representative *Corynebacterium* strains, including 110 validly named species (104 type strains), 15 species with effectively published names and a novel *Corynebacterium* sp. that is pathogenic to yellow-eyed penguins (Saunderson et al., 2021) were obtained from GenBank (Supplementary Table 1). The data included two *Corynebacterium diphtheriae* strains, the type strain DSM 44123^T^ and the extensively studied NCTC 13129 strain. We also included both available assemblies of the type strains of *Corynebacterium imitans*, *Corynebacterium jeikeium*, *Corynebacterium pilosum* and *Corynebacterium renale*, and three assemblies of the type strain of *Corynebacterium minutissimum*. One representative each from the two lineages of *Corynebacterium ulcerans* (Subedi et al., 2018) was included. Type strains of *Corynebacterium hadale* and *Corynebacterium godavarianum*, both heterotypic synonyms of *Corynebacterium gottingense*, were included (Bernard et al., 2020) along with a ‘*Corynebacterium crenatum*’ strain that is considered to be a subspecies of *Corynebacterium glutamicum* (Man et al., 2016). The genome sequences of the type strains of *Corynebacterium accolens* and *Corynebacterium segmentosum*, which were recently found to belong to the same species, were also included (Sagerfors et al., 2021). We also included the type strain of *Corynebacterium xerosis* ATCC 373 isolated from the ear discharge of a child (Bernard and Funke, 2015) and strain GS1 isolated from a caseous nodule from liver of a yak (Wen et al., 2019). Information on the presence of mycolic acids and on host and clinical relevance (importance, source of isolation) was obtained from Bernard and Funke (2015) or the original citations accessed from the LSPN database (Supplementary Table 1).^1^

### Genomic Analyses

The quality of genome assemblies was assessed using CheckM (Parks et al., 2015). All genome sequences that showed more than 90% completeness and less than 5% contamination were considered to be suitable for analysis. These assemblies were automatically annotated using Prokka v 1.13 (Seemann, 2014) and were compared using Roary v 3.12 (Tange, 2011; Page et al., 2015). A protein sequence alignment of 131 core proteins present across all 140 genomes was used to generate a maximum-likelihood tree using IQ-tree v 1.6 using 100,000 ultrafast bootstraps and 100,000 SH-aLRT tests (Nguyen et al., 2015) after removing sites with gaps. Phylogenomic clades with two or more genomes and an average distance from nodes to their leaves <0.15 were assigned a group designation and the remaining strains were treated as singletons.

Protein BLAST searches (Camacho et al., 2009) of 22 gene products from the FAS-I and FAS-II pathways from *M. tuberculosis* strain H37Rv (Table 1) were carried out with an e-value cut-off of 1×10^−5^ to determine the presence of these genes among *Corynebacterium* strains. Much of the extant knowledge relating to mycolic acid biosynthesis has been drawn from a large body of experimental evidence from various bacterial models including, among others, *M. tuberculosis*, *Mycolicibacterium smegmatis* and *Corynebacterium glutamicum*. As the corynebacteria are known to produce short chain-length ‘corynomycolates’, they would be expected to lack genes encoding fatty acid synthase-II (FAS-II), which extends the meromycolate components of mycolic acids in other genera. In order to detect any atypical strains of *Corynebacterium* that might possess FAS-II, we chose query sequences from the well-studied *M. tuberculosis* H37Rv strain; there is strong precedent for significant homology and often synteny when comparing the genetics of multiple aspects of cell wall biosynthesis in corynebacterial genomes with this model.

**Table 1:** List of mycobacterial genes involved in mycolic acid biosynthesis.

System	Gene	Locus	Gene (bp)	Uniprot Accession	Description
	*acpS*	Rv2523c	393	p0a4w8	Holo-[acyl-carrier protein] synthase; 4′-phosphopantetheinyl transferase
FAS-I	*fas-I*	Rv2524c	9,210	p95029	Fatty acid synthase
	*pptT*	Rv2794c	684	O33336	4′-phosphopantetheinyl transferase
	*fabH*	Rv0533c	1,008	p9wng3	β-Ketoacyl-[acyl-carrier-protein] synthase
FAS-II	*fabD*	Rv2243	909	p63458	Malonyl Coa-acyl carrier protein transacylase
FAS-II	*acpM*	Rv2244	348	p9wqf3	Meromycolate extension acyl carrier protein
FAS-II	*kasA*	Rv2245	1,251	p9wqd9	β-Ketoacyl-[acyl-carrier protein] synthase 1
FAS-II	*kasB*	Rv2246	1,317	p9wqd7	β-Ketacyl-[acyl-carrier protein] synthase 2
FAS-II	*accD6*	Rv2247	1,422	p9wqh5	Acetyl/propionyl-Coa carboxylase (beta subunit)
FAS-II	*fabG1*	Rv1483	744	p9wgt3	β-Ketoacyl-[acyl-carrier protein] reductase
FAS-II	*inhA*	Rv1484	810	p9wgr1	NADH-dependent enoyl-[acyl-carrier-protein] reductase
FAS-II	*hadA*	Rv0635	477	p9wfk1	β-Hydroxyacyl-acp dehydratase subunit
FAS-II	*hadB*	Rv0636	429	p96927	β-Hydroxyacyl-acp dehydratase subunit
FAS-II	*hadC*	Rv0637	501	p9wfj9	β-Hydroxyacyl-acp dehydratase subunit
MA condensation	*accD4*	Rv3799c	1,569	o53578	Biotin-dependent long chain acyl-amp carboxylase beta4 subunit
MA condensation	*pks13*	Rv3800c	5,202	o53579	Polyketide synthase
MA condensation	*fadD32*	Rv3801c	1914	o53580	Long-chain-fatty-acid-amp ligase
MA condensation	*accD5*	Rv3280	1,647	p9wqh7	Biotin-dependent acetyl−/propionyl-coenzyme a carboxylase beta5 subunit
MA condensation		Rv3281	534	p96886	Conserved hypothetical protein
MA condensation	*accA3*	Rv3285	1803	p96890	Bifunctional protein acetyl−/propionyl-coenzyme a carboxylase (α-chain)
MA condensation	*accE* ^*^		249		Biotin-dependent acetyl−/propionyl-coenzyme A carboxylase ε subunit
MA reduction	*cmrA*	Rv2059	807	i6y9i3	Dehydrogenase (putative oxidoreductase)

*MA, mycolic acid*.

* *accE gene was used from Corynebacterium glutamicum*.

Duplicate hits with multiple queries were removed and the single best hit with the highest score was retained. We also excluded the hits with sequence identity below 30%. These exclusions were reconsidered where significant absences were noted. The presence of a gene and additional copies (homologues) were manually curated based on the query coverage and sequence identity (Supplementary Table 2). The secondary hits for the protein Rv3285 (encoded by *accA3*) where query or target coverage was <75%, and proteins were 100 amino acids (aa) smaller or larger in size were excluded (Supplementary Table 2). Some hits for the Rv1483 protein (encoded by *fabG1*) were significant but were excluded by reciprocal BLAST searches. In some cases, the presence of conserved gene clusters was considered in prioritising equivocal identifications.

## Results

### *Corynebacterium* Strains and Clinical Importance

The *Corynebacterium* strains studied herein were isolated from diverse sources: 71 from humans, 32 from other mammals and reptiles (antelopes, bharal, cattle, dogs, rodents, seal, tapir, tortoise, etc.), 12 from birds (storks, eagle, geese, ibis and penguins), 20 from environmental sources (including coral, cosmetic dye, fermentation starter, fuel cell, heather, lubricant, river water, marine sediment, sand, soil and sewage) and five from food (banana and dairy products; Supplementary Table 1).

Corynebacterial strains of 54 species that were isolated from humans are clinically important, i.e., are associated with various infections/conditions, two are listed as commensals and two are considered opportunistic human pathogens. *Corynebacterium ulcerans* and *Corynebacterium kutscheri* strains are often isolated from animals and can cause zoonotic infections among humans. Thirteen corynebacterial species are reported to be pathogenic to animals, particularly *Corynebacterium pseudotuberculosis*, *Corynebacterium renale*, *Corynebacterium cystitidis*, *Corynebacterium endometrii*, *Corynebacterium ulceribovis*, *Corynebacterium capitovis*, *Corynebacterium camporealensis* and *Corynebacterium mastitidis* that are pathogenic to cattle and other farm animals (Supplementary Table 1). However, some of these species, such as *Corynebacterium pseudotuberculosis* and *Corynebacterium mastitidis*, can also infect humans (Peel et al., 1997). Therefore, a large proportion of corynebacterial species are either directly associated with infections in humans and animals or are opportunistic pathogens (Supplementary Table 1).

### Phylogenomic Diversity

The average size of the corynebacterial genomes is 2.6 Mb, with the smallest genome reported for *Corynebacterium caspium* (1.8 Mb) and largest for *Corynebacterium glyciniphilum* (3.6 Mb). These genomes are annotated with 1,630–3,316 coding sequences (Supplementary Table 1). Similarly, the GC content is highly variable between the *Corynebacterium* species, varying between 46.5 mol% for *Corynebacterium kutscheri* to 74.7 mol% for *Corynebacterium sphenisci*; overall the mean GC content was 61.3 mol%. The number of rRNA genes varies between 2 and 21 among these genomes with an inverse correlation with the number of contigs: nine or more rRNA genes were identified in 81/84 (96%) genome assemblies with up to five contigs, whereas 51/56 (91%) of assemblies with six or more contigs have 2–7 rRNA genes (Supplementary Table 1). Draft genomes with multiple contigs have lower numbers of rRNA genes annotated, potentially caused by assembly errors due to the repetitive nature of rRNA genes. Likewise, the number of tRNA and tmRNA sequences varied between 45 and 64 and 1 and 2, respectively (Supplementary Table 1).

**Figure 2:**
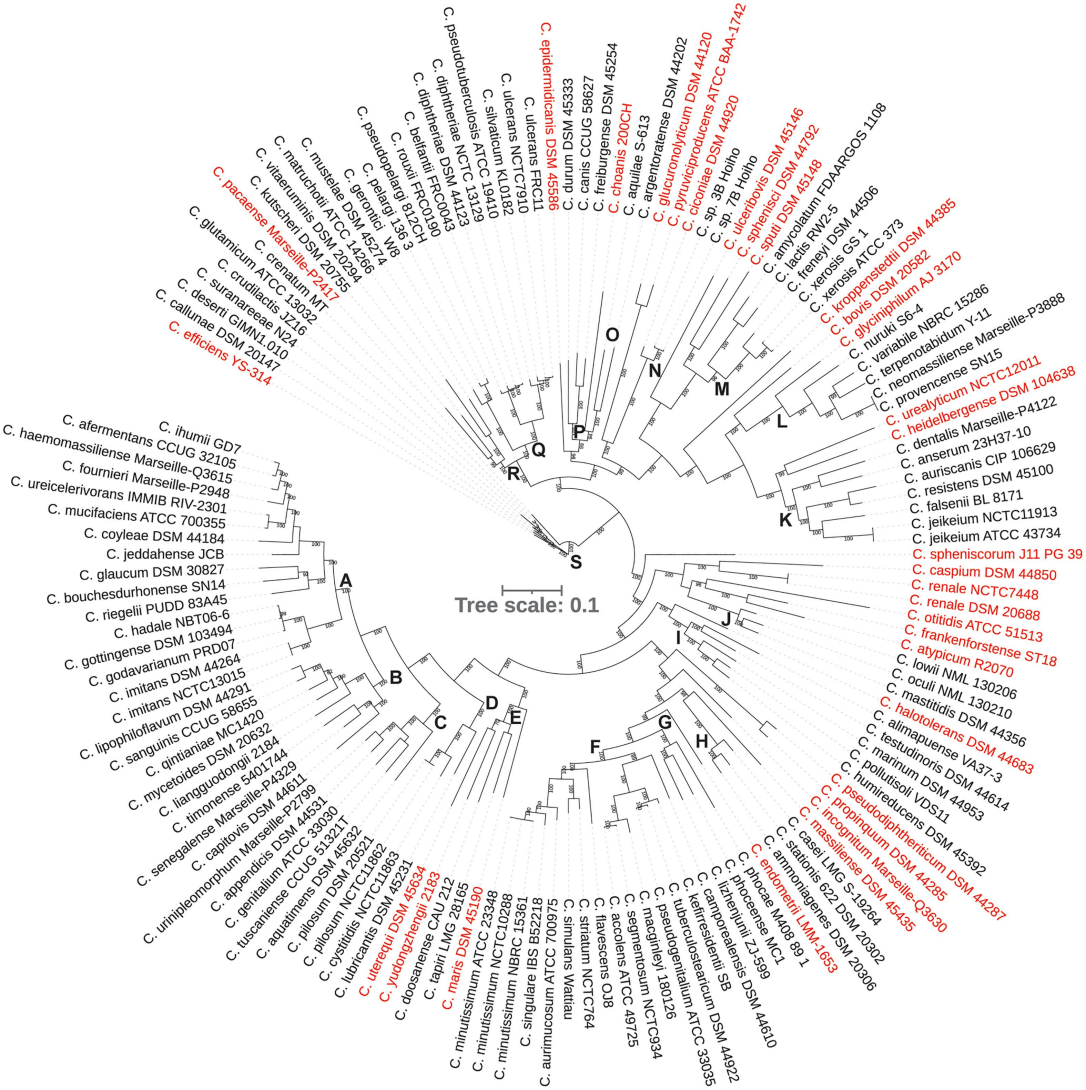
A phylogenetic tree from concatenated protein sequence alignment. The scale bar represents amino acid substitution per site. Singleton strains are shown in red.

A comparative analysis revealed a large and open pangenome of 114,775 genes within the dataset with 112,680 (98%) genes identified as the ‘cloud’ genes, i.e., those present in ≤15% of the genomes in the dataset, 1846 shell genes (present in 15–95% of the genomes), 74 soft core genes (present in 95–99% of the genomes) and 175 were identified as core that were present among 99–100% of the genomes. Of the 175 core genes, 131 genes were present among all 140 strains and protein sequences of these genes were concatenated for phylogenetic analyses. *Corynebacterium* isolates were defined into 19 clades (groups A–S) and 30 species-level singletons that did not group with any other strains (Figure 2). Both the independent genome assemblies of the type strain of *Corynebacterium renale*, associated with infection in cattle grouped together and were treated as a singleton (Figure 2; Supplementary Table 1).

**Figure 3:**
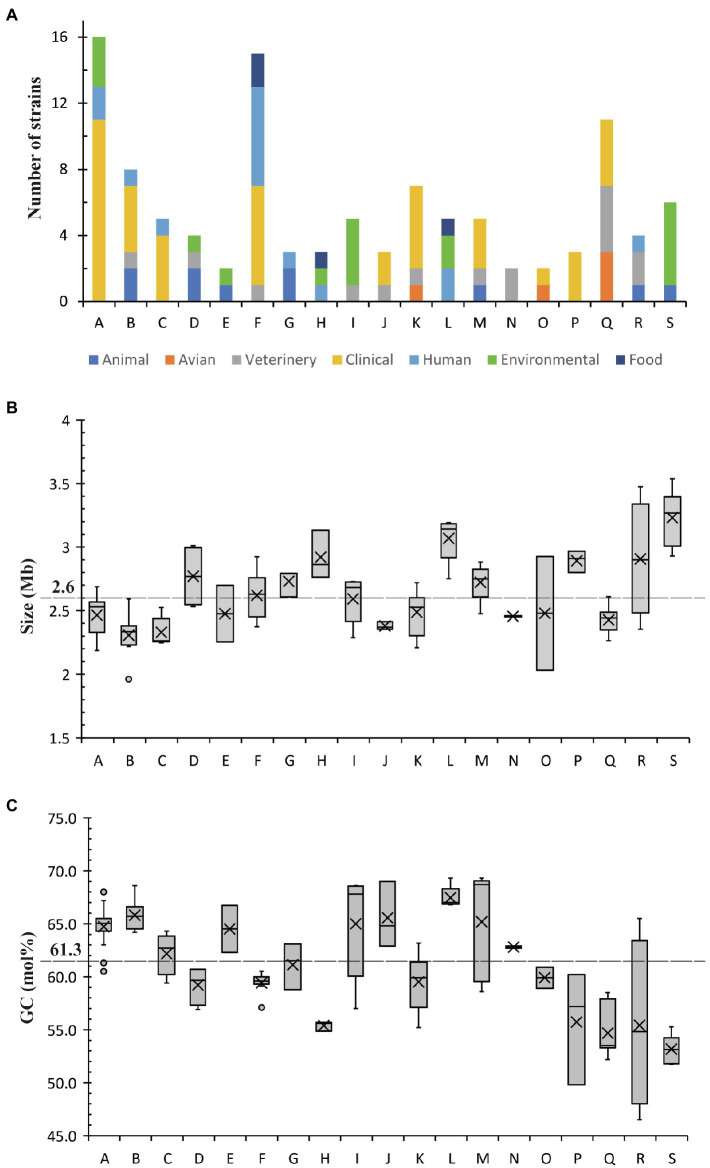
The distribution of **(A)** strains from different sources, **(B)** genome sizes and **(C)** GC content among different phylogenetic groups. Average size and GC content within the genus are highlighted.

Nine major phylogenetic groups (A, B, C, F, I, K, L, Q and S) encompassed five or more species (Figure 2). Although group M contained five strains, two of them were assemblies of *Corynebacterium xerosis* strains, and hence, it was considered as a minor group (Supplementary Table 1). More than 50% of the strains in groups A, B, C, F and K were isolated from humans, mostly associated with clinical infections (Figures 2, 3A; Supplementary Table 1). All five species in group C were isolated from humans with clinical associations (Supplementary Table 1; Figure 3A) except for *Corynebacterium urinipleomorphum*, which was isolated from urine of an infant with rotavirus gastroenteritis (Niang et al., 2019). *Corynebacterium urinipleomorphum* strains have also been isolated from other clinical sources, e.g., from a patient with gallbladder infection along with other bacterial species (Backert et al., 2018). Most of the important human and animal pathogens, including *Corynebacterium diphtheriae*, *Corynebacterium ulcerans*, *Corynebacterium silvaticum*, *Corynebacterium pseudotuberculosis* and *Corynebacterium rouxii*, are grouped in clade Q along with *Corynebacterium pelargi*, *Corynebacterium pseudopelargi* and *Corynebacterium gerontici*. The latter three species were isolated from avian hosts but were not associated with clinical infections (Figure 2; Supplementary Table 1). Approximately 80% of strains in group I and S were isolated from environmental sources. The type strain of *Corynebacterium testudinoris*, clustered in group I, was isolated from necrotic oral lesions in a tortoise along with *Escherichia coli*, a *Streptococcus* species and a *Pseudomonas* species strain; the role of this strain in the infection is unclear (Collins et al., 2001). Group S includes the industrially important species *Corynebacterium glutamicum* and ‘*Corynebacterium crenatum*’ (Ikeda and Takeno, 2013; Man et al., 2016; Kallscheuer and Marienhagen, 2018), originally isolated from sewage and soil, respectively. *Corynebacterium* strains in group L were isolated from diverse sources (human, food or soil) without any clear clinical associations (Figures 2, 3A; Supplementary Table 1).

Among the minor groups, most of human isolates in groups M, J, O and P were from clinical sources. *Corynebacterium freiburgense* and *Corynebacterium canis* strains in group P were isolated from patients’ wounds caused by dog bites and are likely canine in origin (Supplementary Table 1). Three of the four strains in group R are of animal origin, two associated with infections (Figure 3A; Supplementary Table 1). *Corynebacterium kutscheri* was isolated from a rodent but can cause infection in humans (Supplementary Table 1). The four strains in group D belong to three species, two potential pathogens *Corynebacterium pilosum* and *Corynebacterium cystitidis* isolated from bovine hosts, and *Corynebacterium lubricantis* which was isolated from a coolant lubricant (Supplementary Table 1). The close phylogenetic relatedness of human clinical and non-clinical strains in some groups potentially indicates that *Corynebacterium* strains from the latter sources may be able to cause opportunistic infections in humans. Thirty strains designated as singletons were scattered around the tree and were isolated from animals, environment, food and human samples including clinical isolates.

Most of the groups with a higher proportion of clinical strains (except for groups M and P) have genome sizes below the genus average (2.6 Mb; Figure 3B; Supplementary Table 1), whereas the genome size of strains in environmental group S are above the genus average. In contrast, Group I is notable in that most of the isolates are environmental yet have with genome sizes ranging closely around the genus average (2.3–2.7 Mb). There is no clear association between the distribution of GC content and the clinical or environmental source of isolation of phylogenetic clades (Figure 3C; Supplementary Table 1).

Consistent with the recent re-classification of *Corynebacterium hadale* and *Corynebacterium godavarianum* as *Corynebacterium gottingense* (Bernard et al., 2020), all three strains clustered very closely in the phylogenomic tree as did strains of *Corynebacterium accolens* and *Corynebacterium segmentosum* (Sagerfors et al., 2021; Figure 2). Similarly, independent assemblies of the genomes of the type strains of *Corynebacterium imitans*, *Corynebacterium jeikeium*, *Corynebacterium minutissimum*, *Corynebacterium pilosum* and *Corynebacterium renale* were indistinguishable in the core genome tree (Figure 2), demonstrating reproducibility and robustness of the genome sequencing and compilation by different laboratories.

### Mycolic Acid Biosynthesis

Based on the original species descriptions, mycolic acids are absent from the cell envelopes of six corynebacterial species: *Corynebacterium amycolatum*, *Corynebacterium caspium*, *Corynebacterium ciconiae*, *Corynebacterium kroppenstedtii*, *Corynebacterium lactis* and *Corynebacterium otitidis* (Supplementary Table 3). *Corynebacterium atypicum* was originally reported to be lacking mycolic acid based on chemotaxonomic characterisation (Hall et al., 2003) but was found to produce mycolic acids by another study (Wiertz et al., 2013). Of our panel, 85 *Corynebacterium* species were reported to produce mycolic acids; no information was available on the remaining 34 species (Supplementary Table 3).

### Notable Gene Absences in Meromycolate Extension

**Figure 4:**
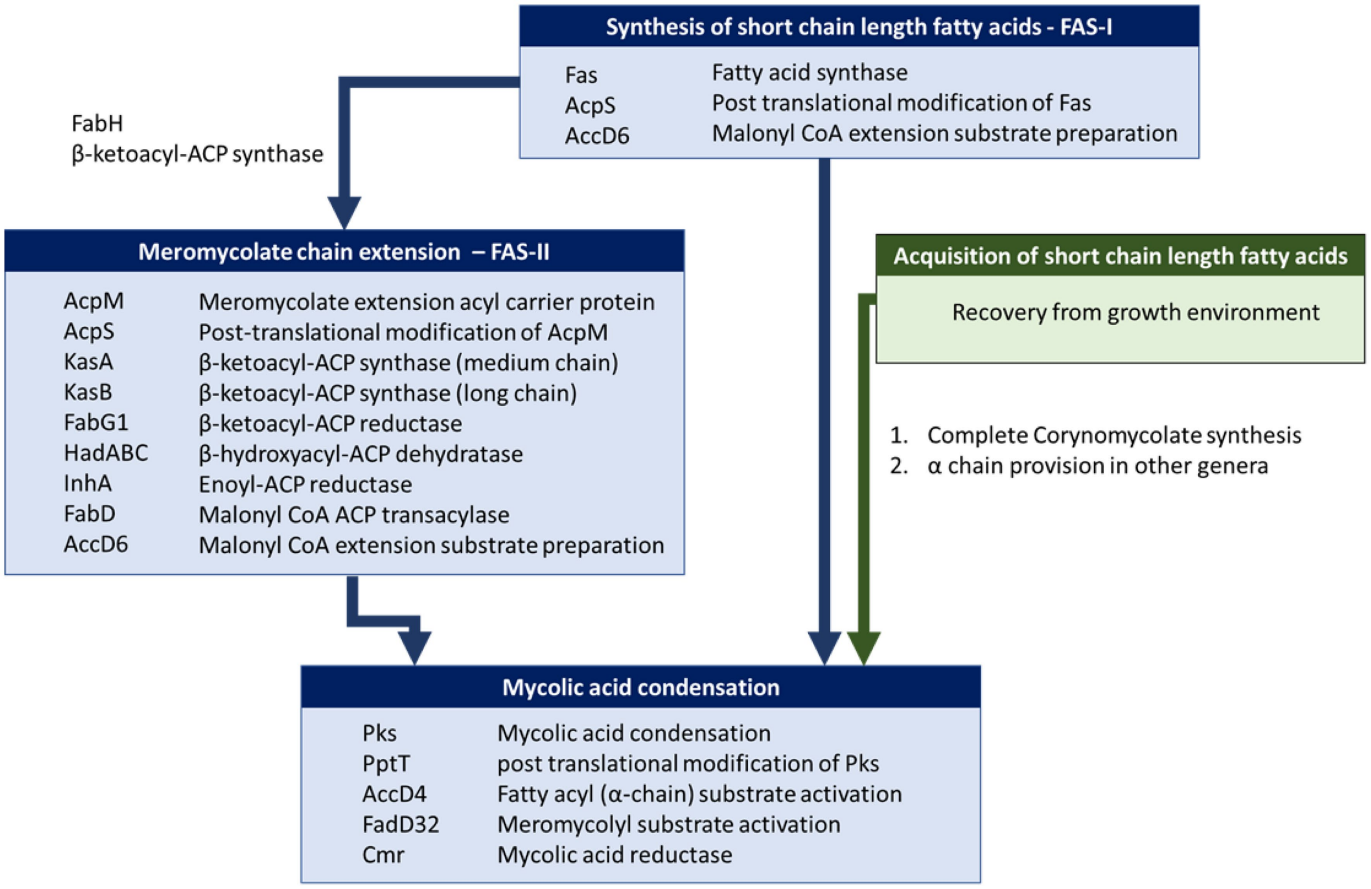
Mycolic acid biosynthetic pathway in *Mycobacterium* and *Corynebacterium*. Briefly, mycolic acid biosynthesis proceeds *via* the activation and condensation of two fatty acyl chains (key enzymes indicated in lower blue box) that ultimately contribute the meromycolyl and α-alkyl chains (see Figure 1). Corynebacteria are unusual in that they do not extend the meromycolyl chain *via* fatty acid synthase II (FAS-II, key enzymes indicated in middle blue box), but rather condense two short fatty acyl chains that may be generated metabolically *via* Fatty Acid Synthase I (key enzymes indicated in upper blue box) or taken up from their habitat (route highlighted in green).

We used BLASTP to identify homologues of proteins involved in mycolic acid biosynthesis. For consistency and to detect atypical strains that might possess FAS-II, we used a set of query sequences derived from *M. tuberculosis* H37Rv. Of the proteins involved in mycolic acid biosynthesis in *M. tuberculosis* (Table 1; Figure 4), Rv0533c (FabH), Rv2243 (FabD), Rv2244 (AcpM), Rv2245 (KasA), Rv2246 (KasB), Rv1483 (FabG1/MabA), Rv1484 (InhA), Rv0635 (HadA), Rv0636 (HadB), Rv0637 (HadC) and Rv3281 (AccE5) were absent among all *Corynebacterium* strains (Supplementary Table 2). Most of these gene absences are expected as they encode components of FAS-II which provides for extended meromycolate chains characteristic of some genera. Similarly, FabH links the FAS-I and FAS-II systems (Brown et al., 2005; Marrakchi et al., 2014). All descriptions of mycolic acid lengths in *Corynebacterium* are consistent with the genus lacking the FAS-II that elongates the meromycolate chain. Our genomic analysis is in complete accord with others in this respect (Dover et al., 2004, 2007; Marrakchi et al., 2014).

### Carboxylase Complexes for Providing Fatty Acid and Polyketide Extension Substrates and Activating the α-Chain Component for Mycolate Synthesis

**Figure 5:**
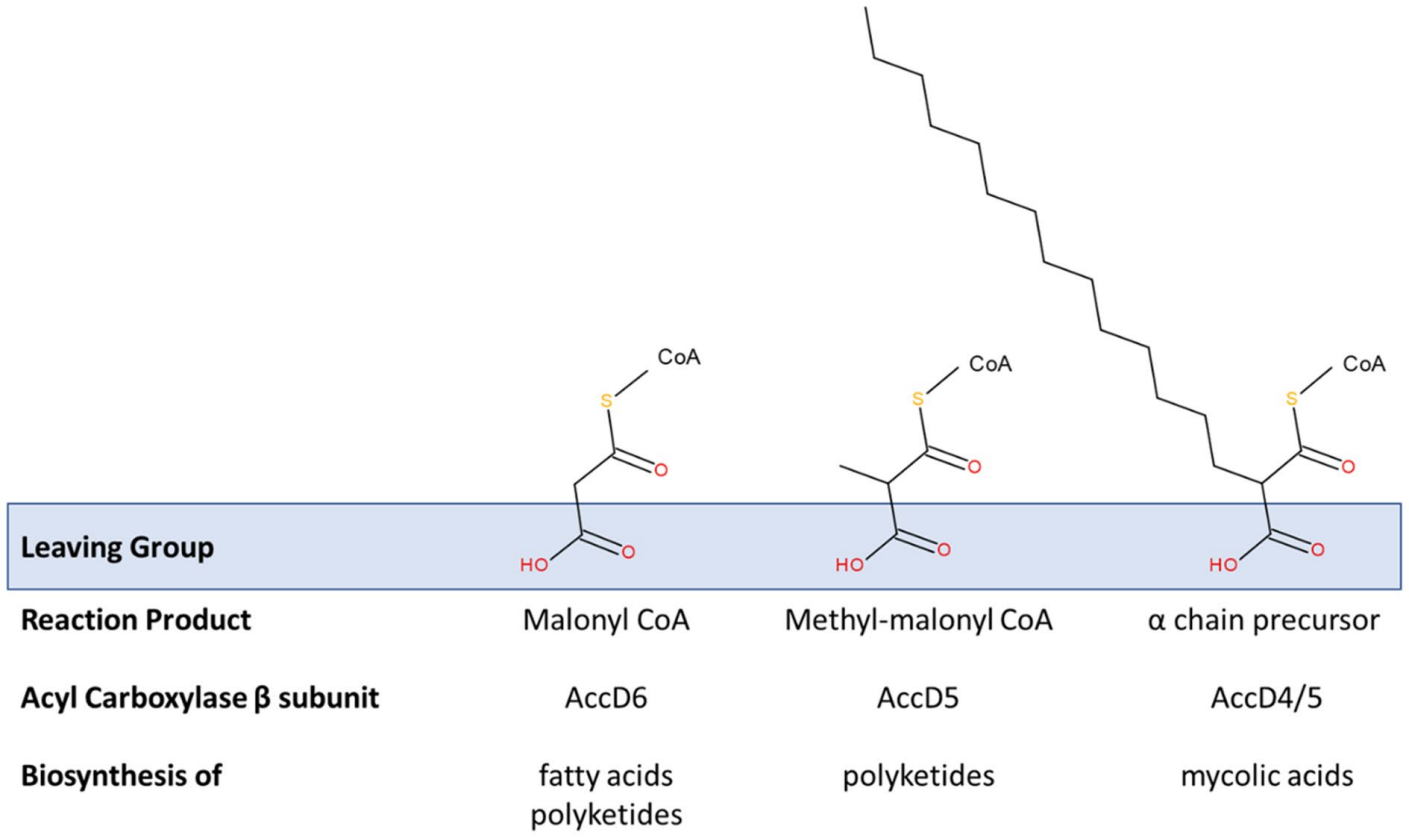
Modification of acyl carboxylase activity by β subunit recruitment. Three acyl carboxylase activities are relevant to the production of fatty acids, polyketides and mycolic acids in studied species of *Mycobacteriales*. All of these are founded on a common α-subunit (AccA3) in a complex with various β-subunits. Those containing the β-subunits AccD4 or AccD5 also contain an ε subunit (AccE5). These acyl carboxylases essentially activate suitable acyl primers to generate substrates for these key biosynthetic processes. A common theme is their involvement in decarboxylative condensation reactions; the carboxyl group is added to promote these reactions and acts as a suitable leaving group (see blue box) that exposes a reactive carbanion that drives the synthetic reaction. The sequence homology shared by these specificity-defining β-subunits is extensive. Their individual functions have been defined through a combination of complex reconstitution and mutagenesis studies. AccD6 prefers an acetyl CoA primer *in vitro* and the carboxylation reaction provides malonyl CoA, an extension substrate used in the synthesis of fatty acids (including *de novo* fatty acid synthesis by FAS-I and meromycolyl chain extension by FAS-II) and polyketides. Acyl carboxylase reconstituted with AccD5 and AccE5 prefers a propionyl CoA primer and forms methyl-malonyl CoA which is used in the synthesis of polyketide molecules. The extent of these substrate preferences may vary and appears to influence the conditional essentiality of AccD6; i.e., acyl carboxylase containing AccD5 may be able to generate enough malonyl CoA to support fatty acid biosynthesis in the absence of *accD6*. AccD4 (likely supported by AccD5 in a heterologous β-subunit complex and AccE5) is responsible for the activation of the α-chain precursor to enable the mycolic condensation (also see Figure 4).

Critical Claisen-like condensation reactions drive the extension of polyketides, fatty acids (including meromycolates) and the condensation of the α and meromycolate chains of mycolic acids. These share a decarboxylative mechanism that requires a substrate with a carboxylic acid leaving group (Figure 5). In mycobacteria, three members of the AccD family of acyl coenzyme A carboxylase β subunits have been associated with fatty acid and polyketide synthesis (including mycolic acid). These are AccD4, AccD5 and AccD6 and these can each form a variety of heterooligomeric complexes with AccA3 and sometimes AccE5 to adapt the catalytic specificity of the acyl carboxylases (Gago et al., 2006).

The biotinylated α subunit of these acyl carboxylases, AccA3, is conserved among all strains and in some cases, strains possessed up to two additional α subunit genes (Supplementary Tables 2 and 3). The simplest substrate generated by these complexes is malonyl CoA which is used in the extension of fatty acids and polyketides by a C_2_H_4_ unit. Malonyl CoA is formed by the complex that contains AccA3 and AccD6 (Rv2247) using an acetyl-CoA primer (Dover et al., 2004; Marrakchi et al., 2014). Some polyketides incorporate methyl-branches by inclusion of a C_3_H_7_ unit derived from methyl-malonyl CoA. AccD5 appears to favour carboxylation of propionyl CoA over acetyl CoA *in vitro*, indicating its primary role may be in producing methyl-malonyl CoA. Although its deletion has been shown to impact upon mycolic acid production, it is likely that it plays a secondary role in mycolic acid biosynthesis.

The gene encoding AccD4 is part of the *accD4*-*pks13*-*fadD32* gene cluster. This acyl carboxylase component is required for the carboxylation of FAS-I products that ultimately form the α-chain in the mycolic acid condensation reaction (Gande et al., 2007). Complexes of AccD5 and AccD4 with AccA3 require the participation of AccE, as presumably this allows for the efficient binding of the larger and branched substrates.

There is extensive homology between each of these β subunits, especially between AccD5 and AccD6, which is consistent with their interactions with the common acyl carboxylase components and their similar substrates. The AccD6 (Rv2247) was found to be absent among *Corynebacterium* strains based on the BLAST similarity criteria (Supplementary Table 2). However, multiple AccD5 homologues were detected and most of them also with significant sequence similarities to AccD6 (Rv2247) and so one of these copies may substitute for AccD6 or participate in other carboxylation reactions. Therefore, we believe that most *Corynebacterium* strains possess between two and four AccD5/AccD6 homologues (Supplementary Tables 2 and 3).

It is conceivable that corynebacteria may be able to satisfy the need for both malonyl and methyl-malonyl extension substrates through only one of these proteins. Both homologues were shown to be able to carboxylate acetyl and proprionyl primers *in vitro* (Daniel et al., 2007) and Pawelczyk et al. demonstrated that, in some mycobacteria, the ability of the AccD5 complex to utilise acetyl CoA as well as proprionyl CoA can influence the essentiality of *accD6* (Pawelczyk et al., 2017).

We could not detect a homologue of the acyl carboxylase ε subunit AccE5 using the *M. tuberculosis* query but were aware from previous studies that an orthologue was present in *Corynebacterium glutamicum* (Gande et al., 2007). Therefore, we used the *Corynebacterium glutamicum* AccE as a query and found that an AccE homologue could be detected in the majority of strains and often clustered alongside an AccD5 homologue, as in *M. tuberculosis*. As the ε subunit appears to be relevant to reactions with branched extension substrates (i.e., with AccD4 and AccD5) and considering the synteny in *M. tuberculosis*, we provisionally assigned the *accD* gene neighbouring *accE* as *accD5*. However, AccE was absent in 19 strains representing 17 species reported to make mycolic acids; two possible explanations are apparent, either this carboxylase subunit is not necessary for corynomycolate biosynthesis in all corynebacteria or the protein exhibits such sequence variation that all orthologues were not detectable using the *Corynebacterium glutamicum* query. This latter scenario is plausible when we consider the small size of the subunit and that the *M. tuberculosis* query detected no homologues despite the robust detection of other gene products.

AccD4 is present among all the corynebacterial strains examined, except for three species *Corynebacterium otitidis*, *Corynebacterium lactis* and *Corynebacterium kroppenstedtii* that do not produce mycolic acids. An additional copy of AccD4 is present in *Corynebacterium ulceribovis*, *Corynebacterium sputi* and *Corynebacterium epidermidicanis*.

### Mycolic Condensation

Alongside AccD4, the gene products of *pks13* and *fadD32* (Rv3801c) form a mycolic condensation system, where FadD32 activates long-chain fatty acids (Le et al., 2016), which are transferred to the phosphopantetheine arm of PKS13 for the final condensation step (Portevin et al., 2004; Gavalda et al., 2014). Subsequently, CmrA (Rv2509) reduces the β-keto group of the product from the mycolic condensation to produce mature mycolic acids, i.e., α-alkyl, β-hydroxy fatty acids (Bhatt et al., 2008; Javid et al., 2020). Additionally, the *pptT* gene product (phosphopantetheine transferase, Rv2794c) is also involved in post-translational modification of type-I polyketide synthases including PKS13 and is thus essential for its activity (Chalut et al., 2006). This gene is also present in most of the strains studied with two copies in ‘*Corynebacterium neomassiliense*’ but is absent from three strains *Corynebacterium amycolatum*, *Corynebacterium lactis* and *Corynebacterium otitidis* (Supplementary Table 3) that do not produce mycolic acids. Some species, for example, *Corynebacterium choanae* and “*Corynebacterium neomassiliense*” have two homologues of FadD32 (Supplementary Table 3).

As expected, four of the six corynebacterial species reported to lack mycolates are missing crucial genes for mycolic acid biosynthesis (Supplementary Table 3), as observed previously for *Corynebacterium amycolatum*, *Corynebacterium kroppenstedtii*, *Corynebacterium lactis* and *Corynebacterium otitidis* (Tauch et al., 2008a; Baek et al., 2018). However, whereas these four genomes lack the *fadD32-pks13-accD4* operon, this locus is present in *Corynebacterium caspium* and *Corynebacterium ciconiae*. Interestingly, an AccE homologue was not detected in *Corynebacterium caspium*; however, we refer to our earlier comments on the reliability of AccE detection. Given these caveats, we consider that *Corynebacterium caspium* and *Corynebacterium ciconiae* are likely to be capable of mycolic acids synthesis, as suggested previously (Baek et al., 2018). Intriguingly, *Corynebacterium bovis*, *Corynebacterium fournieri*, and one of the two *Corynebacterium xerosis* genomes apparently lacks *pks13* gene. *Corynebacterium bovis* has previously been noted to produce distinctively short alkyl-branches (C6-C8; (Collins et al., 1982), whilst no analysis for mycolic acids was carried out when *Corynebacterium fournieri* was described (Diop et al., 2018).

### Provision of Fatty Acyl Precursors for Mycolic Condensation

In *Corynebacterium* strains, *de novo* fatty acid biosynthesis is carried out by Fatty Acid Synthase I. Interestingly, 40 strains belonging to 35 species have an additional copy of *fas* gene (Supplementary Table 3), as previously reported for *Corynebacterium glutamicum* (Radmacher et al., 2005) where one is essential despite the apparent redundancy. Surprisingly, 29 corynebacterial species (30 strains) lack the *fas* gene including 27 that also lack the proximal *acpS* gene responsible for the essential post-translational modification of FAS-I with phosphopantetheine (Table 2). As discussed below, many of these species are reported to be lipophilic, suggesting that these strains must acquire fatty acids from their habitat. Included in this group are all the reported mycolate-lacking strains.

**Table 2:** Presence of mycolic acid biosynthetic genes among corynebacteria, where the Fas protein is absent.

Strain	Lipo^1^	MA^2^	AcpS	CmrA	PptT	AccD5/6	AccA3	AccD4	PKS	FadD32	AccE
*Corynebacterium accolens* ATCC 49725	Yes	Yes									
*Corynebacterium afermentans* CCUG 32105	Yes	Yes									
*Corynebacterium appendicis* DSM 44531	Yes	Yes									
*Corynebacterium aquatimens* DSM 45632	Yes	Yes									
*Corynebacterium bovis* DSM 20582	Yes	Yes									
*Corynebacterium fournieri* Marseille-P2948											
*Corynebacterium jeddahense* JCB											
*Corynebacterium jeikeium* ATCC 43734	Yes	Yes									
*Corynebacterium jeikeium* NCTC11913	Yes	Yes									
*Corynebacterium kroppenstedtii* DSM 44385		No									
*Corynebacterium lipophiloflavum* DSM 44291	Yes	Yes									
*Corynebacterium lowii* NML 130206	Yes										
*Corynebacterium macginleyi* 180,126	Yes	Yes									
*Corynebacterium mastitidis* DSM 44356	Yes	Yes									
*Corynebacterium oculi* NML 130210	Yes										
*Corynebacterium pyruviciproducens* ATCC BAA-1742	Yes	Yes									
*Corynebacterium resistens* DSM 45100	Yes										
*Corynebacterium segmentosum* NCTC934											
*Corynebacterium tuberculostearicum* DSM 44922	Yes	Yes									
*Corynebacterium tuscaniense* CCUG 51321	No	Yes									
*Corynebacterium urealyticum* NCTC12011	Yes	Yes									
*Corynebacterium ureicelerivorans* IMMIB RIV-2301	Yes	Yes									
*Corynebacterium xerosis* ATCC 373	No	Yes									
“*Corynebacterium bouchesdurhonense*” SN14											
“*Corynebacterium dentalis*” Marseille-P4122											
“*Corynebacterium genitalium*” ATCC 33030											
“*Corynebacterium heidelbergense*” DSM 104638	Yes	Yes									
“*Corynebacterium kefirresidentii*” SB											
“*Corynebacterium pseudogenitalium*” ATCC 33035											
“*Corynebacterium urinipleomorphum*” Marseille-P2799											

*An absence of protein is shown in white, presence in green, two copies in blue and three copies of a protein is shown in pink. The effectively published names are mentioned in quotation marks. Purple, see section “Discussion” for consideration of these genomes*.

1 *Lipophilic*.

2 *Mycolic acids reported*.

Overall, 82 genomes belonging to 75 species have all mycolic acid biosynthetic genes present, whilst in 24 genomes (21 species) we detected all genes except AccE, including 19 strains from 17 species that are reported to produce mycolates. Thirty-four genomes (32 species) have at least one key missing gene involved in fatty acid and/or mycolic acid biosynthesis (Table 2; Supplementary Table 3). Of those 32 species, 12 (13 strains) that lack Fas (10 also lacking AcpS) without further absences are likely to be able to generate mycolic acids using exogenous fatty acids. Notably, mycolates have been reported in 8 of these species. Similarly, additional 12 species (13 strains) that lack Fas/AcpS with AccE undetected are also likely to be able to make mycolates from exogenous fatty acids, as reported for six of these species. Five species lacking some or all of the *accD4*, *fadD32* and *pks13* genes are likely to be unable to produce corynomycolates. Some of these genes also appears to be absent in *Corynebacterium atypicum*, *Corynebacterium bovis* and *Corynebacterium xerosis*, potentially due to the assembly/annotation errors, which is discussed below.

## Discussion

### Genomic Diversity and Virulence

It is well-known that the genus *Corynebacterium* comprises species of industrial, medical or veterinary importance, in addition to human and animal commensals (Bernard and Funke, 2015; Sangal and Burkovski, 2020). Currently, 133 validly named species (excluding synonyms) are listed in the LSPN database (https://lpsn.dsmz.de/genus/corynebacterium; accessed on September 30, 2021). In this study, we have analysed the genome sequences of a collection of corynebacterial genomes with a good representation of the species diversity (126 species, including 15 with effectively published names and one unnamed species from yellow-eyed penguins) from diverse sources including animals, environment, food and humans (Supplementary Table 1). Interestingly, more than 50% of these species (69/126) are associated with clinical conditions/symptoms, including opportunistic infections in humans and/or animals (Supplementary Table 1). Whilst *Corynebacterium diphtheriae*, *Corynebacterium ulcerans* and *Corynebacterium pseudotuberculosis* are the most notable pathogenic species within the genus, this study highlights the true scale of the pathogenic potential of corynebacteria, both for humans and animals, which has not been previously appreciated.

*Corynebacterium* is very diverse phylogenomically; this study defines 19 clades with a further 30 singleton strains (Figure 2; Supplementary Table 1). With respect to the range of genome sizes reported, the largest is 2-fold larger than the smallest with a proportionate number of coding sequences (*Corynebacterium caspium*: 1.8 Mb and 1,630 CDS and *Corynebacterium glyciniphilum*: 3.6 Mb and 3,316 CDS) and their GC contents vary from 46.5 mol% for *Corynebacterium kutscheri* to 74.7 mol% for *Corynebacterium sphenisci* (Supplementary Table 1). Most of the groups with a higher proportion of clinical isolates have smaller genome sizes (with a few exceptions; Figure 3A), which may indicate reductive genome evolution associated with adaptation to a pathogenic lifestyle (Weinert and Welch, 2017). Interestingly, all bar one of the major clades (i.e., those with >5 strains) include isolates from multiple animal hosts/sources including those of clinical origin. The exception is group S, where most strains have an environmental origin. Group Q includes human pathogens, such as *Corynebacterium diphtheriae* and *Corynebacterium rouxii*, and animal pathogens, such as *Corynebacterium ulcerans* and *Corynebacterium pseudotuberculosis*. The latter species are also able to infect humans. Therefore, this indicates the possibility that other isolates from non-human species may also be able to infect humans.

### Mycolic Acid Biosynthesis in Corynebacteria

The analysis for the presence of genes involved in mycolic acid biosynthesis among corynebacteria has been informative (Figure 4). As expected, the data confirm an absence of FAS-II biosynthetic pathway within the genus *Corynebacterium* (Dover et al., 2004, 2007; Marrakchi et al., 2014). The presence of all the other genes recognised as essential for mycolic acid biosynthesis is demonstrated among strains of 67 species that are reported to produce mycolic acid and additional 24 species where phenotypic data is not available (Supplementary Table 3). The study revealed the absence of key genes in *Corynebacterium amycolatum*, *Corynebacterium kroppenstedtii*, *Corynebacterium lactis* and *Corynebacterium otitidis* that is consistent with the documented absence of corynomycolates in their cell envelopes (Supplementary Table 3). *Corynebacterium fournieri* may be an additional corynebacterial species that lacks mycolates as PKS is not detected. Conversely, all required genes for mycolic acid biosynthesis are present in *Corynebacterium ciconiae* but it is reported to lack mycolic acids (Bernard and Funke, 2015), which potentially reflect errors in phenotypic characterisation.

We also noted 15 species that are phenotypically reported to produce mycolates but lack FAS-I (Table 2). Most of these species are reported to be lipophilic (except for *Corynebacterium tuscaniense*; Table 2; Supplementary Table 1) with AcpS protein also being absent. AcpS is associated with the crucial phosphopantetheinyl post-translational modification of FAS-I (Chalut et al., 2006; Gokulan et al., 2011), a function which is no longer needed if the latter is missing. Thus, it is likely that corynebacterial strains with the *fas* and *acpS* genes missing, are able to use exogenous fatty acids to synthesise mycolic acids, unless additional gene deletions in the mycolate biosynthesis pathway are present (as in *Corynebacterium kroppenstedtii* and *Corynebacterium otitidis*). Of the 29 corynebacterial species (30 strains) that lack the *fas* gene, 18 are reported to be lipophilic (Table 2). The corelation between lack of FAS-I and lipophilism provides a clear explanation for the origin of this phenotype, as suggested previously from observations on selected species (Tauch et al., 2008a,b; Tauch and Burkovski, 2015). Interestingly, *Corynebacterium godavarianum*, *Corynebacterium sanguinis*, *Corynebacterium endometrii* and *Corynebacterium sputi* strains are also reported to be lipophilic but possess *acpS* and *fas* genes (Supplementary Table 3). As mentioned before, *Corynebacterium godavarianum* has been reclassified as *Corynebacterium gottingense* and the latter is reported to be non-lipophilic and produces mycolates. These discrepancies may reflect difference in culture and/or methodology for assessing lipophilism.

This study also reports the absence of multiple genes including *fas* and *pks13* in the type strain of *Corynebacterium xerosis* ATCC 373 whereas all mycolic acid biosynthesis genes are present in another isolate of this species, GS1 (Supplementary Table 3). *Corynebacterium xerosis* ATCC 373 has been reported to produce mycolic acids (Collins et al., 1982; Bernard and Funke, 2015). Two smaller putative proteins mapped partially on PKS13 and four on parts of FAS-I (data not shown), potentially indicating that both the genes are pseudogenes or the multiple gaps (poor/incomplete genome quality) likely contributed to the discrepancy with phenotypic characterisation of mycolates in this strain. Similarly, despite the apparent absence of PKS13 in *Corynebacterium bovis*, we found two smaller genes, GCA_000183325.2_01024 (encoding 71 aa) and GCA_000183325.2_01025 (encoding 319 aa), showing significant similarities with parts of PKS13. This genome has 503 contigs and the higher number of gaps may be responsible for only partial detection of this gene, given it is reported to make mycolic acids (Collins et al., 1982). *Corynebacterium atypicum* R-2070 (=DSM 44849) may lack *fadD32* (Supplementary Table 3), although it is noted that Tippelt et al. (2014) reported a complete mycolate biosynthesis pathway in *Corynebacterium atypicum* DSM 44849 (Tippelt et al., 2014), so the absence of *fadD32* noted here may reflect the use of different annotation pipelines. The thin-layer chromatographic identification of mycolates in the study of Wiertz et al. (2013) is also consistent with this conclusion and suggests that the original description of this species as lacking mycolates is incorrect.

This study also shows a presence of second copy of the *fas* gene in 35 corynebacterial species but there is no obvious association to any phylogenetic group (Supplementary Table 3). Two copies of *fas* gene, *fasA* and *fasB*, have been characterised in *Corynebacterium glutamicum* where *fasA* was shown to be the essential fatty acid synthase, and the non-essential *fasB* has a supplementary role. Although deletion of *fasB* gene did alter the cornomycolate profile, it was shown to be associated with supplying palmitate and enhanced growth of the bacterium (Radmacher et al., 2005). Deletion of *fasA* resulted in fatty acid auxotrophy which is consistent with our hypothesis that loss of this gene creates the lipophilic phenotype among *Corynebacterium* strains.

### Role of Mycolic Acids in Virulence

Mycolic acids in the mycobacterial cell envelope are known to help bacteria in resisting environmental stresses such as antibiotics and in virulence by manipulating the host immune system (Korf et al., 2005; Dao et al., 2008; Vander Beken et al., 2011; Marrakchi et al., 2014; Nataraj et al., 2015). However, the effects of the more complex mycobacterial mycolates on virulence are primarily linked to modifications (such as cyclopropanation; Barkan et al., 2012) that are not features of the simpler corynomycolates (Figure 1).

Early work demonstrated that lipid extracts from *Corynebacterium pseudotuberculosis* had cytotoxic effects on mouse macrophages, but these phenomena were host dependent and the effects were not observed with guinea pig or rabbit macrophages (Hard, 1975). Trehalose mycolate glycolipids from non-pathogenic *Corynebacterium glutamicum* can also activate mouse macrophages (Chami et al., 2002) and this family of glycolipids are well-established as immunopotentiators (Schick et al., 2017; Burkovski, 2018). Intradermal injection of extracts containing corynomycolates from *Corynebacterium pseudotuberculosis* induced mild histopathological lesions in female goats (Jesse et al., 2020) but these extracts may also contain other immunogenic lipids. Interestingly, *Corynebacterium diphtheriae* strains delayed the maturation of phagolysosomes after internalisation in murine and human cell lines, but this was also observed with a strain which lacks corynomycolates (Ott et al., 2017). In addition, induction of cytokines appears to be a mycolate-independent mechanism in *Corynebacterium diphtheriae* strains (Ott et al., 2017; Schick et al., 2017). However, the mycolate-free strain showed increased susceptibility to several classes of antibiotics, consistent with the role of mycolates in presenting an outer membrane permeability barrier.

Notably, several corynebacterial species that lack mycolic acid biosynthetic genes are human clinical isolates (Supplementary Table 3). The loss of *pks13* in *Corynebacterium glutamicum* did not affect the viability of the strain but compromised the growth rate and the ability to grow at temperatures above 30°C (Portevin et al., 2004). Therefore, we suggest that the presence of mycolic acids in the cell envelope is a fundamental structural feature that affects strain fitness and helps them to resist environmental stresses such as antibiotic action but is not directly involved in virulence.

## Data Availability Statement

The original contributions presented in the study are included in the article/Supplementary Material, further inquiries can be directed to the corresponding author.

## Author Contributions

VS conceived the study. VS, LD, and IS contributed to the design of the study and were involved in data interpretation and drafting of the manuscript. LD, AT, and VS performed data analyses. All authors contributed to the article and approved the submitted version.

## Conflict of Interest

The authors declare that the research was conducted in the absence of any commercial or financial relationships that could be construed as a potential conflict of interest.The reviewer AB declared a past collaboration with one of the authors VS to the handling editor.

## Publisher’s Note

All claims expressed in this article are solely those of the authors and do not necessarily represent those of their affiliated organizations, or those of the publisher, the editors and the reviewers. Any product that may be evaluated in this article, or claim that may be made by its manufacturer, is not guaranteed or endorsed by the publisher.

## Footnotes

^1^ https://lpsn.dsmz.de/genus/corynebacterium
